# Changes in occupational well-being during COVID-19: the impact of age, gender, education, living alone, and telework in a Finnish four-wave population sample

**DOI:** 10.5271/sjweh.4033

**Published:** 2022-08-31

**Authors:** Janne Kaltiainen, Jari Hakanen

**Affiliations:** 1 Finnish Institute of Occupational Health, Työterveyslaitos, Finland, Helsinki

**Keywords:** burnout, Finland, job boredom, longitudinal work engagement, population study, within-person change

## Abstract

**Objectives:**

This study investigated how occupational well-being evolved across different phases, before and during the COVID-19 outbreak in the Finnish population. Whereas studies have suggested that certain demographic groups (eg, young, female) are more at risk during COVID-19, less is known whether the effects of such demographic factors may vary (i) across different phases of the unfolding viral outbreak and (ii) on different dimensions of occupational well-being. As they are predictors of changes in burnout, job boredom, and work engagement, we examined age, gender, education, living alone, and teleworking. This is the first study to provide such detailed knowledge regarding the changes in various occupational well-being dimensions before and during the COVID-19 outbreak.

**Methods:**

We collected randomized population panel data at the end of 2019 and conducted three follow-up surveys with 6-month intervals (N=532). The data were analyzed with latent change score models.

**Results:**

Whereas during spring 2020, occupational well-being slightly improved, in autumn 2020 well-being decreased back to pre-COVID-19 levels. There was an indication of slight increases in job boredom between before COVID-19 and summer 2021. Well-being deteriorated more for the young and those who lived alone. There was also some indication of females, those with lower education, and non-teleworkers experiencing less favorable changes in occupational well-being. Teleworking appeared to have more beneficial effects on well-being for those with lower education.

**Conclusions:**

The study suggests that only relatively minor changes in well-being took place among the employed population. A particular focus in workplaces should be targeted at younger employees.

COVID-19 brought about changes for millions of employees, which may have subsequently led to changes in occupational well-being during the pandemic. Substantial changes in work arrangements include an increase in teleworking, which occurred especially during the early stages of the pandemic (1). By teleworking we refer to working outside the conventional or central workplace (eg, from home) with the aid of technology to interact with others (2). Importantly, the impact of COVID-19 has varied depending on the profession, which may be reflected in how occupational well-being changed for males and females (3). For instance, the impact for IT professionals may have arguably been different from those who work in social and healthcare. Also, the well-being of the young and those who live alone may have been especially endangered (4) given the importance of social interactions for these groups, which has been severely restricted during the COVID-19. It may also be that those who are more familiar with teleworking arrangements (ie, those with higher education) may experience the forced and typically full-time teleworking during COVID-19 differently than those for whom teleworking is potentially a more novel aspect of work (ie, those with lower education) thus resulting in differing well-being changes. Despite the growing number of studies examining the effects of COVID-19, to the best of our knowledge, none have examined how different dimensions of occupational well-being have evolved during these exceptional times on a population level and whether there are differences in such changes depending on age, gender, education, living alone, and teleworking across timespans characterized with different contextual circumstances.

Notably, to provide a more holistic understanding regarding employee well-being, it is essential to examine both positive states – ie, work engagement as a positive affective-motivational state characterized by vigor, dedication, and absorption at work (5) – and ill-being at work. Furthermore, as regards ill-being at work, it is important to distinguish between states stemming from overstimulation, ie, burnout, from states that typically stem from understimulation, ie, job boredom. Whereas burnout refers to a state of exhaustion, cynicism, loss of emotional control, and impaired cognitive functioning at work (6), job boredom is a state characterized by low arousal, mind-wandering, and perceptions of slow passage of time (7). Importantly for the study at hand, these different dimensions of occupational well-being may develop independently from each other. For instance, it may be that certain characteristics of the COVID-19 pandemic fostered motivation and positive affectivity at work (eg, work engagement) while simultaneously levels of ill-being (eg, burnout) may have evolved differently. Arguably for some, COVID-19 may have resulted in higher workload, fostering burnout, whereas for others the pandemic may have impoverished work tasks and environments and consequently promoted job boredom.

We examined population data of matched survey respondents collected across 1.5 years, starting from three months before the COVID-19 outbreak, with three follow-up surveys with six-month time intervals. We investigated potential changes in occupational well-being over four stages of the pandemic and whether those changes differed depending on age, gender, education, living alone, and teleworking. We furthermore investigated whether the effect of telework on changes in occupational well-being differed depending on the education level. Occupational well-being is examined via work engagement – a pleasant state with high activation – and via job boredom and burnout, both representing unpleasant states with low activation (8).

## Methods

### Study design, sample, and context

The study design is retrospective and longitudinal. All data were collected via postal and electronic surveys. Approximately three months before the COVID-19 outbreak, in December 2019 and January 2020, the first survey was posted to 2609 individuals who were randomly chosen from the registry of Finnish residents aged 18–65 years. Altogether 517 surveys were completed (response rate of 19.8%). The electronic survey was sent to 6366 individuals who were randomly selected from an existing online panel consisting of 30 000 Finnish citizens. Of those, 1136 responded (response rate of 17.8%). Taloustutkimus Inc, an established research company in Finland, collected the data as instructed by the authors. The Ethical Review Committee of the Finnish Institute of Occupational Health approved the study (records 7/2019, 4/2020, 9/2020, and 5/2021).

After excluding those who were not employed or returned a survey with a substantial amount of missing information, there were 1567 eligible respondents at Time 1 (T1). At Time 2 (T2), in June and July 2020, 1076 responded to the first follow-up survey (response rate 68.6%). At Time 3 (T3) in December 2020 and January 2021, 823 responded (76.5%). Finally, at Time 4 (T4), between May and July 2021, 615 responded (74.7%). After excluding those who either were not employed at all time points or worked <10 hours a week (N=82) and those who did not provide information about their education level (N=1), the final analyzed sample included 532 individuals who responded to all four surveys. Of these, 21.1% (N=112) responded via postal survey and 78.9% (N=420) via electronic survey. The response method (internet or postal survey) was not statistically significantly associated with changes in any of the occupational well-being dimensions across any of the examined time spans (please contact the first author for detailed results). The sample description is given in table 1.

**Table 1:** Characteristics of all participants at Time 1 (N=532). [SD=standard deviation]

	Unweighted	Weighted	Unweighted
		
	Mean (SD)	Mean (SD)	N (%)
Age (years)	47.28 (10.10)	44.56 (11.43)	
19–25			7 (1.3)
26–35			70 (13.2)
36–45			137 (25.8)
46–55			190 (35.7)
56–65			128 (24.1)
Gender (value)	0.60 (0.49)	0.52 (0.50)	
Male (0)			213 (40.0)
Female (1)			319 (60.0)
Education level (value)	1.99 (0.77)	1.97 (0.79)	
Low (1)			161 (30.3)
Intermediate (2)			214 (40.2)
High (3)			157 (29.5)
Lived alone (value)	0.29 (0.46)	0.29 (0.45)	
No (0)			356 (66.9)
Yes (1)			148 (27.8)
Missing			28 (5.3)
Teleworking (value)	0.38 (0.49)	0.34 (0.47)	
Non-telework (0)			245 (62.2)
Telework (1)			149 (37.8)
Missing			138 (25.9)
Work contract (value)	0.08 (0.28)	0.11 (0.32)	
Permanent (0)			485 (91.2)
Temporary (1)			45 (8.5)
Missing			2 (0.4)
Supervisory/management position (value)	0.15 (0.36)	0.14 (0.35)	
No (0)			450 (84.6)
Yes (1)			81 (15.2)
Missing			1 (0.2)
Work time a week (hours)	37.94 (5.82)	37.83 (6.01)	
Occupational group			
Worker			203 (38.2)
Clerical worker			103 (19.4)
Upper clerical worker, expert			175 (32.9)
Management			25 (4.7)
Entrepreneur			23 (4.3)
Student			2 (0.4)
Missing			1 (0.2)
Employment sector (value)	1.70 (0.66)	1.70 (0.67)	
Public (1)			209 (39.3)
Private (2)			281 (52.8)
Other (3)			42 (7.9)
Industry sectors			
Municipal sector			123 (23.1)
Manufacturing and industry			72 (13.5)
Government			57 (10.7)
Wholesale and retail sale			34 (6.4)
Business services			34 (6.4)
Trusts and associations			31 (5.8)
Construction and energy			24 (4.5)
Transport, traffic			23 (4.3)
Telecommunications, postal			20 (3.8)
Accommodation, catering			14 (2.6)
Engineering, architect, design			14 (2.3)
Finance, insurance			11 (2.1)
Media, advertising			9 (1.7)
Agriculture, forestry, fishing			6 (1.1)
Other			62 (11.7)

We used weighting by age, gender, and residential area in the analyses to match the population distribution. At T1, the means of the weight coefficients were statistically significantly larger for males (mean 1.84) than females [mean 1.24, F(1,1565)=124.57, P<0.001]. Age correlated negatively with the weight coefficient (R=−0.483, P<0.001). The means of weight coefficients were 5.48 for those who were ≤25 years old, and 2.06, 1.33, 1.21, and 1.12 for those who were 26–35, 36–45, 46–55 and ≥56 years old, respectively. For residential areas, the weight coefficient means were 1.25 for Helsinki-Uusimaa, 1.53 for other parts of Southern Finland, 1.55 for Western Finland, and 1.88 for North and Eastern Finland with statistically significant differences between these groups [F(3,1563)=24.36, P<0.001]. These findings suggest that males, the young, and citizens from North and Eastern Finland were less likely to respond at T1. However, by applying the weight coefficients in the analyses, we were able to some extent combat the potential biasing effect of non-response at the baseline.

Attrition analyses over time indicated that those who remained in the sample across the study (N=532), did not differ from those who responded only at T1 (N=1035) in terms of T1 levels of burnout [t(1565)=1.069, P=0.285], job boredom [t(1563)=0.427, P=0.670], work engagement [t(1563)=0.097, P=0.922], and gender [t(1565)=−0.281, P=0.779]. However, those who remained in the sample were slightly older (mean 47.28) than those who responded only at T1 [mean 45.02; t(1565)=−3.876, P<0.001], and were slightly more educated [mean 1.99 versus 1.87; t(1559)=−2.873, P<0.01]. Given that these differences were relatively small, these findings did not indicate that our main results would be substantially affected by the non-random sampling.

The first wave of COVID-19 hit Finland in March 2020, approximately three months after the T1 data collection. This meant substantial changes to millions of employees and citizens. Schools were closed and access to daycare was restricted until 14 May 2020. Based on emergency powers legislation, social gatherings of >10 people became illegal, and restaurants and many public services were closed, and social events were canceled. Also, access to and from the southern part of Finland was restricted by army-enforced checkpoints for three weeks in Spring 2020. Government and health officials strongly recommended teleworking from home to all who were able to do so and refrain from meeting other people face-to-face outside one’s household. This was widely complied with as many employers closed offices. Approximately 61% of Finnish employees switched to teleworking from home, which was the highest number in Europe (9). During the second wave of COVID-19, from September to December 2020, the highest number of 14-day incidence of COVID-19 cases was around 116, whereas during the third wave from February to April 2021, this number was around 174 (10). The official governmental recommendation to telework and various social restrictions continued across the study. However, curfews were not implemented at any time point.

### Predictors

The demographics, including age, were measured at T1. Gender was dichotomized with 0=male and 1=female. Education level was coded into three categories: 1=compulsory, vocational, or upper secondary education (“low”), 2=institute or university of applied sciences (“intermediate”), and 3=university-level education (“high”). Living alone was dichotomized with 0=did not live alone and 1=lived alone at T3 and T4. Here 28 were coded as missing as their living conditions changed between T3 and T4. Teleworking was measured at T2, T3, and T4. Those who did not telework at all across these three time points were given a value of 0 (ie, non-teleworkers) and those who teleworked at least 75% of their working time at all three time points were given a value of 1 (ie, teleworkers). Here 25.9% (N=138) were coded as missing due to not providing information on their teleworking time at all three time points (N=12) or their amount of telework changed across the examined time spans (N=126). Of the teleworking group, N=109 (73.2%) teleworked all their working time across the measured time points and N=134 (90.5%) reported at T2 that their teleworking time had substantially increased since the COVID-19 outbreak.

### Outcomes

*Burnout*. Burnout Assessment Tool (BAT-23; 6) with altogether 23 items including exhaustion (8 items; eg, “At work, I feel mentally exhausted”), cynicism (5 items, eg, “I struggle to find any enthusiasm for my work”), cognitive impairment (5 items, eg, “At work, I struggle to think clearly”), and loss of emotional control (5 items, eg, “At work, I feel unable to control my emotions”). The responses were given on a 5-point scale, 1=never; 2=rarely; 3=sometimes; 4=often; 5=always.

*Job boredom*. Three items were adapted from the Dutch Boredom Scale (7) with items “During work time I daydream”, “At work, time goes by very slowly”, and “I feel bored at my job”, which reflected the three aspects of job boredom; behavioral, cognitive, and affective, respectively. The responses were given on a 7-point scale, 0=never; 1=few times a year; 2=once a month; 3=few times a month; 4=once a week; 5=few times a week; 6=daily.

*Work engagement*. A three-item scale, the Ultra-Short Measure for Work Engagement (UWES-3) (5) tapped into experiences of vigor, dedication, and absorption at work (eg, “At my work, I feel bursting with energy”). The responses were given on a 7-point scale, 0=never; 1=few times a year; 2=once a month; 3=few times a month; 4=once a week; 5=few times a week; 6=daily.

The Pearson correlations between burnout and job boredom were 0.54, 0.53, 0.54, and 0.55 at T1, T2, T3 and T4, respectively. The same correlations between burnout and work engagement were −0.59, −0.58, −0.63, and −0.63. Between work engagement and job boredom these correlations were −0.40, −0.45, −0.44, and −0.42. All correlation estimates were statistically significant at P<0.001.

### Statistical analyses

To examine changes in occupational well-being and the impact of predictors on these changes, we used structural equation modeling (SEM) framework, namely latent change score modeling (LCSM) (11, 12), which is widely used in behavioral and social sciences. The model description is given in the supplementary material, www.sjweh.fi/article/4033. In LCSM, the latent change score represents within-person changes across specific time intervals and allows for testing of statistical significance of mean changes and whether other variables explain between-person variability in such changes (13, 14). Notably, as the latent change scores are construed on latent scores, which are estimated with multiple observed items, the latent change scores are purged of measurement error (15). In contrast, analytical techniques which operate with observed variables (eg, paired t-tests, mean differences, or residual change scores) cannot achieve this and assume perfect measurement reliability (15, 16). Thus, LCSM provides more accurate estimates for the true changes in the outcome variables and the impact of predictor variables on such changes. Another strength of latent variable modeling with multiple indicators is that we can establish that the potential changes in the well-being constructs are not due to the survey items being interpreted differently amongst the participants across time points, which is another potential cause of bias in the parameter estimates in non-latent analytical techniques (15, 17). This measurement invariance is achieved by setting the factor loadings, item intercepts, and item residuals to equal across time and comparing the increasingly constrained models with less constrained models. The over-time invariance was established for all the well-being factors (please contact the first author for detailed results). Taken together, whereas LCSM replicates paired t-test, ANCOVA, and mean difference analyses, it does so in SEM framework and thus does not suffer from the same methodological limitations that change analyses with observed variables do (14, 18, 19). Furthermore, as we examine time-invariant predictors (eg, gender), fixed-effects models are not suitable for our purposes.

The models are estimated separately for each occupational well-being dimension and time span. When testing for moderating effects, the predictor and moderating variables were standardized and the interaction coefficient was calculated by multiplying the predictor variable with the moderating variable, and all three were regressed on outcome variables.

## Results

### Changes in occupational well-being in the full sample

**Figure 1:**
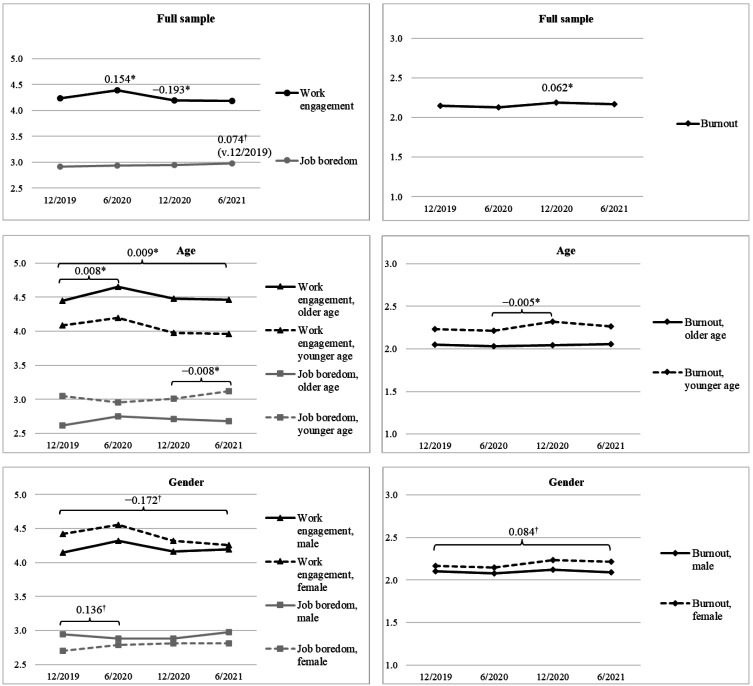
The means and changes in occupational well-being in the full sample (age-, gender-, and education-adjusted 12/2019 observed means and latent changes), for age (gender- and education-adjusted 12/2019 observed means and latent changes), and for gender (age- and education-adjusted 12/2019 observed means and latent changes). Older age as +1SD and younger age as −1SD. Unstandardized coefficients are shown. Only coefficient which 95% (*) or 90% (†) confidence intervals exclude zero are shown.

By examining the age-, gender- and education-adjusted means of latent change scores (Δμ) in the full sample (N=532; see figure 1), we found that work engagement increased across T1–T2 as indicated by the statistically significant and positive mean of latent change score (Δμ=0.154, 95% CI 0.064–0.244). Across T1–T2, there were no changes in job boredom (Δμ=0.019, 95% CI −0.062–0.099) or in burnout (Δμ=−0.020, 95% CI −0.059–0.019). Across the subsequent time span (T2–T3), burnout increased (Δμ=0.062, 95% CI 0.022–0.102) and work engagement decreased (Δμ=−0.193, 95% CI −0.269–−0.117). There were no changes in job boredom across T2–T3 (Δμ=0.011, 95% CI −0.049–0.070). Across the latest time span (T3-T4), there were no changes in burnout (Δμ=−0.025, 95% CI −0.069–0.020), job boredom (Δμ=0.037, 95% CI −0.038–0.112), or work engagement (Δμ=−0.016, 95% CI −0.106–0.073). When comparing T1 and T4 means, there was an indication of slight increases in job boredom (Δμ=0.074, 95% CI -0.001–0.149; 90% CI 0.01–0.137), whereas there were no changes in burnout (Δμ=0.011, 95% CI -0.039–0.062) or in work engagement (Δμ=−0.057, 95% CI -0.155–0.040).

### The impact of age, gender, education, living alone, and telework on changes in well-being

To test the associations between the five predictor variables (ie, age, gender, education, living alone, teleworking) and the outcome variables (ie, changes in three dimensions of occupational well-being across four timespans), we estimated altogether 60 path estimates (see table 2). In the following, we discuss estimates which were either statistically significant at 95% confidence intervals (six estimates) or marginally significant at 90% confidence intervals (eight estimates).

**Table 2:** The estimates from latent change score models regarding the relations from gender, age, education, living alone, and telework to changes in three dimensions of occupational well-being. Standardized path estimates with 95% confidence intervals (CI) are shown. **Estimates with 95% CI excluding zero are bolded.** [T1=Time 1; T2= Time 2; T3=Time 3; T4=Time 4].

Model	Predictor variable	ΔT1-T2 (a)		ΔT2-T3 (b)		ΔT3-T4 (c)		ΔT1-T4 (d)	
			
		Standardized regression coefficient	95% CI	Standardized regression coefficient	95% CI	Standardized regression coefficient	95% CI	Standardized Regression coefficient	95% CI
Outcome variable: Burnout									
M1a-d	Age	-0.002	-0.005-0.001	**-0.016**	**-0.030–−0.001**	0.005	−0.005–0.014	-0.005	-0.015–0.005
	Gender	0.029	-0.041–0.098	0.183	-0.081–0.447	0.085	−0.119–0.289	0.185†	-0.007–0.376
	Education	-0.004	-0.047–0.039	-0.064	-0.230–0.103	-0.088	−0.223–0.047	-0.041	-0.169–0.087
	R_2_	0.006	-0.006–0.019	**0.043**	**0.010–0.076**	0.010	−0.007–0.027	0.014	-0.006–0.034
M2a-d	Living alone ^a^	0.146	-0.064–0.356	0.088	-0.181–0.356	0.034	−0.215–0.284	0.195†	-0.033–0.423
	R_2_	0.008	-0.007–0.023	**0.055**	**0.017–0.093**	0.014	−0.006–0.034	0.021†	-0.004–0.046
M3a-d	Telework ^a^	0.045	-0.218–0.308	-0.270†	-0.559–0.020	0.181	−0.090–0.451	-0.058	-0.303–0.187
	R_2_	0.008	-0.009–0.025	**0.093**	**0.039–0.147**	0.016	−0.008–0.040	0.015	-0.009–0.039
Outcome variable: Job boredom									
M4a-d	Age	0.004	-0.010–0.018	-0.008	-0.018–0.003	**-0.013**	**−0.024– −0.002**	-0.006	-0.016–0.004
	Gender	0.228†	-0.010–0.467	0.033	-0.185–0.250	-0.101	−0.318–0.116	0.109	-0.098–0.316
	Education	0.074	-0.074–0.221	-0.073	-0.209–0.063	0.009	−0.131–0.149	0.058	-0.074–0.190
	R_2_	0.018	-0.004–0.040	0.011	-0.006–0.028	0.024	−0.001–0.049	0.010	-0.007–0.027
M5a-d	Living alone ^a^	**0.258**	**0.029–0.487**	0.118	-0.132–0.367	0.091	−0.165–0.346	**0.272**	**0.025–0.520**
	R_2_	**0.031**	**0.002–0.060**	0.016	-0.006–0.038	0.025†	−0.002–0.052	0.023†	-0.003–0.049
M6a-d	Telework ^a^	0.152	-0.150–0.454	0.116	-0.168–0.400	0.141	−0.143–0.425	0.192	-0.095–0.479
	R_2_	0.026	-0.005–0.057	0.017	-0.008–0.042	0.034†	−0.001–0.069	0.031†	0.003–0.063
Outcome variable: Work engagement									
M7a-d	Age	**0.010**	**0.001–0.018**	0.006	-0.003–0.015	0.003	−0.007–0.012	**0.009**	**0.001–0.018**
	Gender	0.069	-0.125–0.263	-0.086	-0.295–0.123	-0.084	−0.289–0.121	-0.179†	-0.368–0.011
	Education	0.101†	-0.015–0.218	-0.029	-0.162–0.104	0.075	−0.063–0.212	0.069	-0.057–0.195
	R_2_	0.019	-0.004–0.042	0.008	-0.007–0.023	0.006	−0.007–0.019	0.023†	-0.002–0.005
M8a-d	Living alone ^a^	-0.131	-0.327–0.064	-0.088	-0.319–0.143	0.139	−0.106–0.385	-0.031	-0.236–0.174
	R_2_	0.023†	-0.003–0.049	0.013	-0.006–0.032	0.010	−0.007–0.027	0.022†	-0.003–0.047
M9a-d	Telework ^a^	0.220†	-0.032–0.471	0.004	-0.270–0.278	-0.056	−0.303–0.190	0.197†	-0.013–0.407
	R_2_	**0.046**	**0.006–0.086**	0.034†	-0.001–0.069	0.005	−0.009–0.019	0.046	0.006–0.086

† Estimates with 90% confidence interval excluding zero are marked.

Δ Indicates within-person change across specific time span. Each predictor variable is estimated in separate models.

a The model included three additional predictor variables (age, gender, and education) as control variables and path estimates for these control variables are shown in the supplementary material, table S1.

The impact of age, gender, and education (ie, demographic variables) was tested in models in which all these three variables were set to predict changes in occupational well-being (Models M1, M4, and M7 in table 2). As shown in table 2, age was associated with less increases in burnout across T2–T3 (M1b; Table 2) and with less increases in job boredom across T3–T4 (M4c; see also figure 1). Age was also associated with more increases in work engagement across T1–T2 (M7a) and across T1–T4 (M7d).

**Figure 2:**
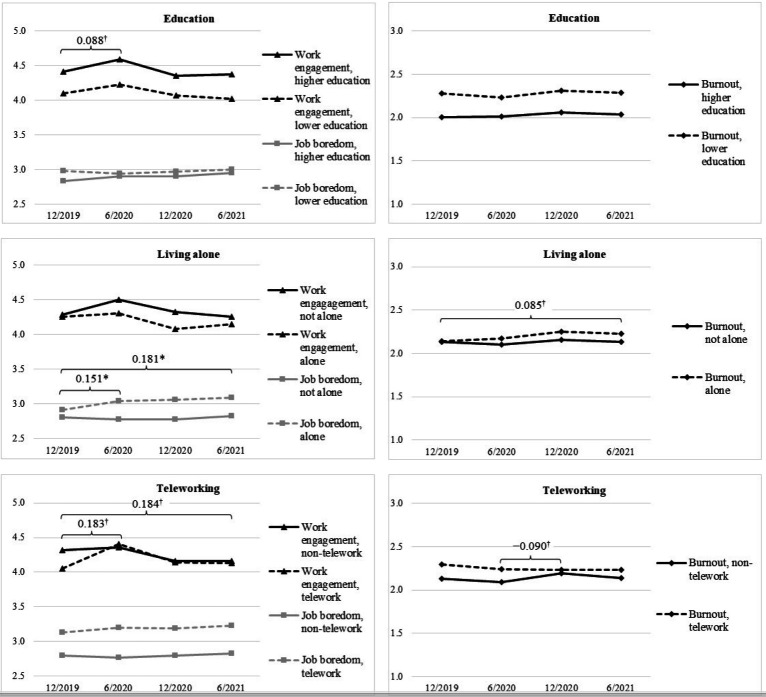
The means and changes in occupational well-being for education (age- and gender-adjusted 12/2019 observed means and latent changes) and for living alone and telework (age-, gender-, and education-adjusted 12/2019 observed means and latent changes). Higher education as +1SD and lower education as −1SD. Unstandardized coefficients are shown. Only coefficients which 95% (*) or 90% (†) confidence intervals exclude zero are shown.

There was an indication of being a female (rather than male) being associated with more increases in burnout across T1–T4 (table 2, M1d, β=0.185, 90% CI 0.024–0.345), more increases in job boredom across T1–T2 (M4a, β=0.228, 90% CI 0.029–0.428), and less increases in work engagement across T1–T4 (M7d, β=−0.179, 90% CI −0.338–−0.020; see also figure 1). Only one of the estimates regarding the effects of education was marginally statistically significant and it indicated that those with higher education experienced slightly more increases in work engagement across T1–T2 (table 2, M7a, β=0.101, 90% CI 0.004 –0.199; see also figure 2).

The effects of living alone and teleworking during COVID-19 were drawn from models in which the effects of age, gender, and education were controlled for (M2, M5, M8 in table 2; see supplementary table S1 for the estimates for the control variables). Living alone during COVID-19 was associated with increases in job boredom across T1–T2 (M5a) and T1–T4 (M5d; see also figure 2). There was also an indication of living alone being related to increases in burnout across T1–T4 (M2d, β=0.195, 90% CI 0.004–0.386).

There was only indication of teleworking being associated with changes in occupational well-being. Teleworking was marginally associated with less increases in burnout across T2–T3 (Table 2, M3b, β=−0.270, 90% CI -0.513– -0.027) and more increases in work engagement across T1–T2 (M9a, β=0.220, 90% CI 0.009–0.430) and across T1–T4 (M9d, β=0.197, 90% CI 0.021–0.373; see also figure 2).

### Interactions between telework and education

**Figure 3:**
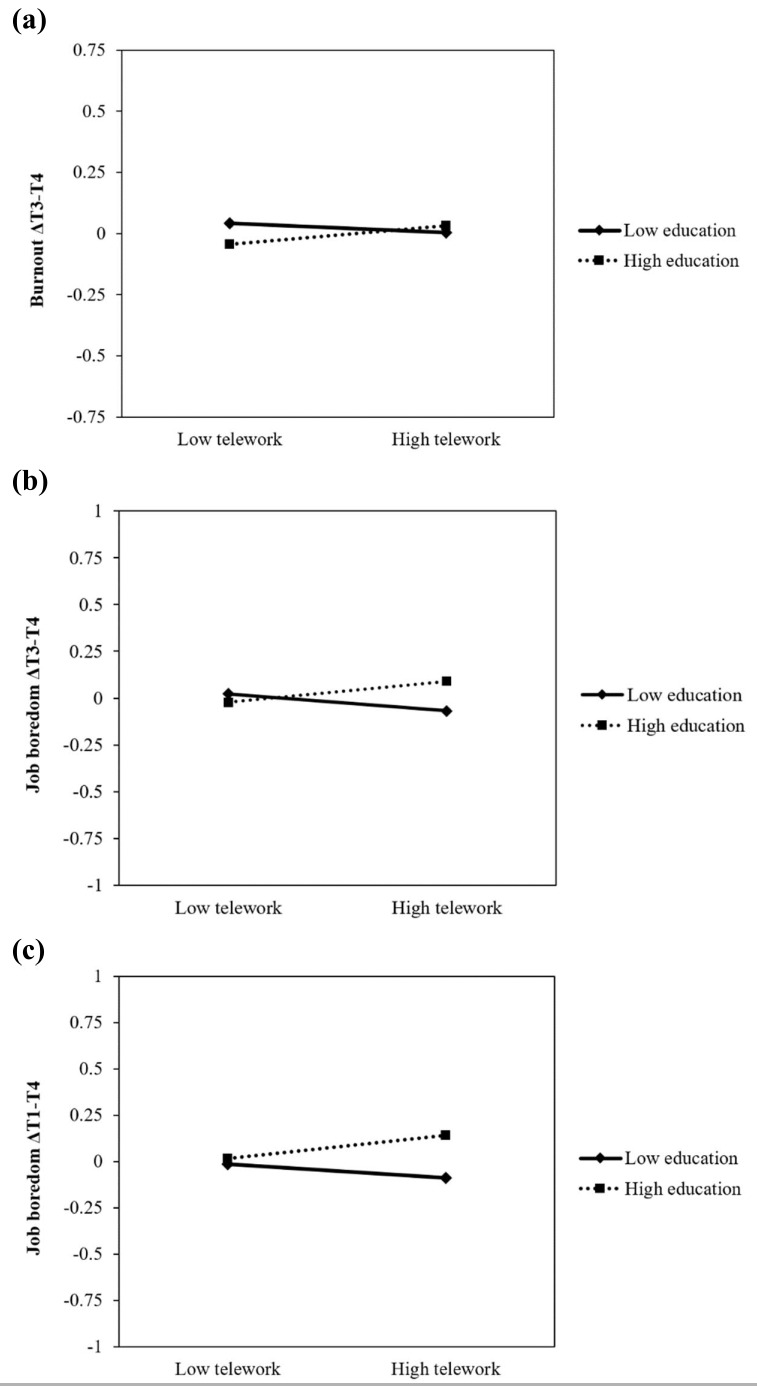
The interactions between (a) telework and education on changes in burnout across T3-T4, (b) telework and education on changes in job boredom across T3-T4, and between (c) telework and education on changes in job boredom across T1-T4, Low and high moderator values are set as +/− 1 SD.

We tested whether education level moderated the relationships between teleworking and changes in occupational well-being. Potentially, those who are more educated may have been more familiar with teleworking before COVID-19, which thus may affect the impact of teleworking on employees’ well-being. Accordingly, we found that the interaction coefficient of teleworking and education was statistically significant when predicting changes in burnout across T3–T4 (β=0.137, 95% CI 0.005–0.270) and changes in job boredom across T3–T4 (β=0.154, 95% CI 0.028–0.280) and T1–T4 (β=0.151, 95% CI 0.031–0.272). As shown in figure 3a, for those with higher education, teleworking was associated with more increases in burnout T3–T4, whereas the opposite was found for those who had lower education. Similarly, teleworking was associated with more increases in job boredom across T3–T4 (figure 3b) and T1–T4 (figure 3c) for those with higher education, whereas for those with lower education, teleworking was associated with less increases in job boredom. The 95% CI of other interaction coefficients for telework and education included a zero (please contact the first author for detailed results).

## Discussion

Our findings show that occupational well-being to some extent improved across the first COVID-19 stage in the Spring of 2020 (T1–T2) as work engagement increased while there were no changes in burnout and job boredom. These results align with other studies reporting favorable changes in occupational well-being during similar time spans (eg, 20, 21). These findings may indicate that at first, the COVID-19 related restrictions implemented in Spring 2020 may have led to some favorable changes in working conditions on average, such as temporarily easing the general intensity at work and non-work and thus improving well-being. This may be especially so for those who switched to teleworking as work engagement increased especially for them (see below). However, by the end of 2020 (T2–T3), occupational well-being deteriorated as work engagement decreased and burnout increased. This finding may indicate that the same restrictions that at first potentially had a favorable impact on well-being (T1–T2), when prolonged, had an unfavorable impact over time. That is, prolonged restrictions that prevented meaningful activities and face-to-face interactions with others likely eventually worsened working conditions, for instance by harming social relations at work, thus deteriorating well-being over the subsequent phase (T2–T3). The stability in occupational well-being found during Spring 2021 (T3–T4) in turn may indicate adjustment to the situation over time and/or less changes in physical and psychosocial working conditions.

When comparing the mean levels before COVID-19 in late 2019 (T1) and summer 2021 (T4), the findings showed that burnout and work engagement did not change, whereas there was an indication that employees experienced job boredom slightly more often in summer 2021 than before COVID-19. Such increases in job boredom may be caused by the impoverishment of work environments, tasks, and social relations which may have occurred due to various social restrictions at work. In general, there were only small changes in occupational well-being during the study. This aligns with findings suggesting that lockdowns during COVID-19 have had only a small effect on mental health (22) and with studies suggesting no substantial changes in general well-being during COVID-19 (eg, 23). It may also indicate that on average at the population level, there were no substantial changes in working conditions. However, the change trends differed within the population sample to some extent.

### The young, females, single households, and non-teleworkers were more at risk

Young age was associated with declining occupational well-being in terms of more increases in burnout during autumn 2020 (T2–T3) and job boredom in spring 2021 (T3–T4). Young age was also associated with less increases in work engagement during spring 2020 (T1–T2) and when comparing pre-COVID-19 levels with summer 2021 (T1–T4). Relatedly, Evans et al (24) found that depression increased amongst young students during spring 2020. These findings may be due to COVID-19 related social restrictions, which are likely to impair social relations at work and subsequently have the strongest negative impact on those who depend most on such social connections. For example, for the young it may be especially important to learn from tacit knowledge, receive easily available immediate support and feedback, and build social networks. Potentially younger employees are also more likely to work in occupations (eg, service sector) in which working conditions were more strongly, and negatively affected by the pandemic (eg, increase in uncertainty). This effect appears to first manifest in decreases in motivation and engagement, which is later followed by other negative outcomes, such as increases in job boredom and burnout. This illuminates how the effect of age was different across differing contextual circumstances during COVID-19 and varied between the examined occupational well-being dimensions.

There was some indication of occupational well-being deteriorating more for females than males. Specifically, there was an indication that females experienced more increases in burnout and less increases in work engagement across the study (T1–T4) and more increases in job boredom during spring 2020 (T1–T2). These findings echo results from other studies suggesting that females’ mental health has deteriorated more during COVID-19 (25, 26). The gender effect may be due to female-dominated occupations (eg, human service jobs, teaching, social and health care) being more directly exposed to the negative consequences of the COVID-19 pandemic (eg, increase in workload, loss of job resources, the fear of COVID-19 infection). For instance, in the study’s sample, 24.4% of males worked in industry and manufacturing and 11.3% in the municipality sector, whereas for females these percentages were 6.3% and 31.0%, respectively. Notably, these marginally significant effects were seen only approximately 1 year and 3 months after the COVID-19 outbreak (T1–T4).

Living alone during COVID-19 was associated with a decline in occupational well-being as job boredom increased for these participants more during spring 2020 (T1–T2) and across the study time span (T1–T4). As an indication, also burnout appeared to increase somewhat more for those who lived alone between late 2019 and summer 2021 (T1-T4). These findings suggest that during times of general social restrictions that likely harm social relations at work, it may be especially important whether one finds social contacts and resources within one’s household.

There was also an indication of non-teleworkers experiencing less favorable changes in occupational well-being in comparison to teleworkers. Specifically, non-teleworkers experienced slightly less increases in work engagement during spring 2020 (T1–T2) and between late 2019 and summer 2020 (T1–T4). There was also an indication of non-teleworkers experiencing more increases in burnout during autumn 2020 (T2–T3). These tentative findings suggest that, overall, teleworking may have supported some of the working conditions, such as introducing new work practices, more autonomy at work, and opportunities to learn new things at work, which are known to foster occupational well-being such as work engagement (eg, 27). At the same time, non-teleworkers are more likely to work in jobs (eg, service industries) that are potentially more affected by the negative consequences of the pandemic such as having face-to-face contacts while such contacts were considered a health risk. Similarly, Ervasti et al (28) found that working from home was associated with better mental health during the pandemic.

Interestingly, the effect of telework on occupational well-being appeared to differ depending on the level of education in some instances. For those with higher education, telework was more likely to be associated with decreases in occupational well-being (ie, increases in burnout and job boredom in autumn 2020, and increases in job boredom between late 2019 and summer 2021). This finding may suggest that perhaps for those with less education, teleworking was associated with more improvements in working conditions, such as increases in new work practices, autonomy, and learning new things at work (ie, aspects of work that benefit occupational well-being) than for those with higher education, who may also be more used to teleworking. Relatedly, Wanberg et al (29) found that education was associated with more increases in depressive symptoms and a greater decrease in life satisfaction during COVID-19, thus suggesting a similar negative effect. Our study suggests that this may especially be the case among the highly educated who worked from home.

Taken together, the change trends did not substantially differ depending on the examined demographic and background variables for instance in terms of explained variance (table 2). One potential conclusion from this is that changes in occupational well-being are more likely explained by changes in the working conditions and subjective experiences (30) than differences in demographics or work arrangements (ie, face-to-face or telework) in the population. Accordingly, studies have suggested that the subjective perception of loneliness is a stronger predictor of well-being than living alone or physical isolation during COVID-19 (31). Yet, the current examination provides important insights into how occupational well-being evolved for different demographic groups, which could be overshadowed in studies focusing solely on working conditions. Furthermore, when predicting processes that occur over time such as within-person changes, typically less variance is explained in comparison to predicting scores at specific time points.

### Strengths and limitations

We acknowledge that with a larger sample size, a larger part of the examined path estimates would have been statistically significant, and the findings could be generalized with stronger confidence. However, we used weighting for the statistical analyses to strengthen the representativeness of our sample and the means of the weight coefficients did not indicate overly large or extreme differences between the sample and population distributions at the baseline. Furthermore, the analyses regarding attrition over time did not indicate that, for instance, those who reported higher occupational well-being would have been more likely to respond also at the final follow-up, which would have introduced bias. Yet, the relatively small sample size and its selection at the baseline and over time limit the generalizability of our findings.

As all the data was collected by self-report surveys in a non-experimental study design, our study provides only limited causal conclusions as some of the examined associations may be impacted by omitted variables. Similarly, given that including a control group who did not experience the COVID-19 pandemic in the study design was not plausible in practice, we cannot test whether the changes or stabilities in occupational well-being are due to COVID-19. Yet, the focal constructs necessitate the use of self-reporting, and our investigation of within-person changes across time with methods accounting for measurement error and invariance strengthen causal inferences to some extent. As working conditions are notable predictors of occupational well-being (27, 32), the exclusion of such measures from this study further limits the inferences we can make regarding the reasons why occupational well-being changed or did not change during the pandemic. Furthermore, data with more frequent measurement time points could have provided more detailed information regarding the potential variability in the change trends and the impact of specific COVID-19 related restrictions. Yet, the current data enabled us to investigate how different dimensions of occupational well-being evolved over four different stages of the COVID-19 pandemic amongst the same participants with a baseline before the COVID-19 outbreak, thus representing a strong contribution to the COVID-19 and occupational well-being literature. We also note that given the large number of estimated coefficients, there is a possibility that some of the statistically significant findings may be due to chance.

Another strength of the study is the examination of three different dimensions of occupational well-being. As shown by the results, the evolution of these dimensions differed from one another. This underlines the importance to examine occupational well-being as a multidimensional phenomenon covering mental states that can be both negative (job boredom, burnout) and positive (work engagement) to provide a more nuanced and holistic understanding on the topic. Furthermore, the correlations between the constructs (see Methods section) did not indicate a substantial overlap between the constructs, thus supporting the theoretical notions and prior empirical findings of work engagement, burnout, and job boredom representing different states of occupational well-being (eg, 5, 32).

### Concluding remarks

Our findings suggest that in general the impact of demographics, living alone, and teleworking have been quite minor for employee well-being during different phases of the pandemic and compared to baseline a few months before the outbreak of the COVID-19. Being employed even during highly exceptional times still comprises many latent functions and fulfills several needs that may sustain well-being, for instance, compared to those without jobs (33). However, particularly the well-being of young employees and those who lived alone have been more at risk during COVID-19. We also suggest for future research to investigate multiple indicators of employee well- and ill-being as it fosters a comprehensive and more nuanced understanding regarding occupational well-being.

### Conflict of interest

The authors declare no conflicts of interest.

## Supplementary material

Supplementary material
