# Supplementary material for: Changes in occupational well-being during COVID-19: the impact of age, gender, education, living alone, and telework in a Finnish four-wave population sample
Source: Scand J Work Environ Health. 2022 Aug 31;48(6):457–67. doi: 10.5271/sjweh.4033 (PMC9888443; doi:10.5271/sjweh.4033)
Supplement: Supplementary material [file SJWEH-48-457-S001.pdf]

Changes in occupational well-being during COVID-19: the impact of age, gender, education, living alone, and telework in a Finnish 4-wave population sample[1]

by Janne Kaltiainen, DSocSci,[2] Jari Hakanen, DSocSci

1. Supplementary material
2. Correspondence to: Janne Kaltiainen (Corresponding author), Doctor of Social Science, Finnish Institute of Occupational Health, P.O. Box 40, FI-00032 Työterveyslaitos, Finland, Helsinki. [E-mail: janne.kaltiainen@ttl.fi]

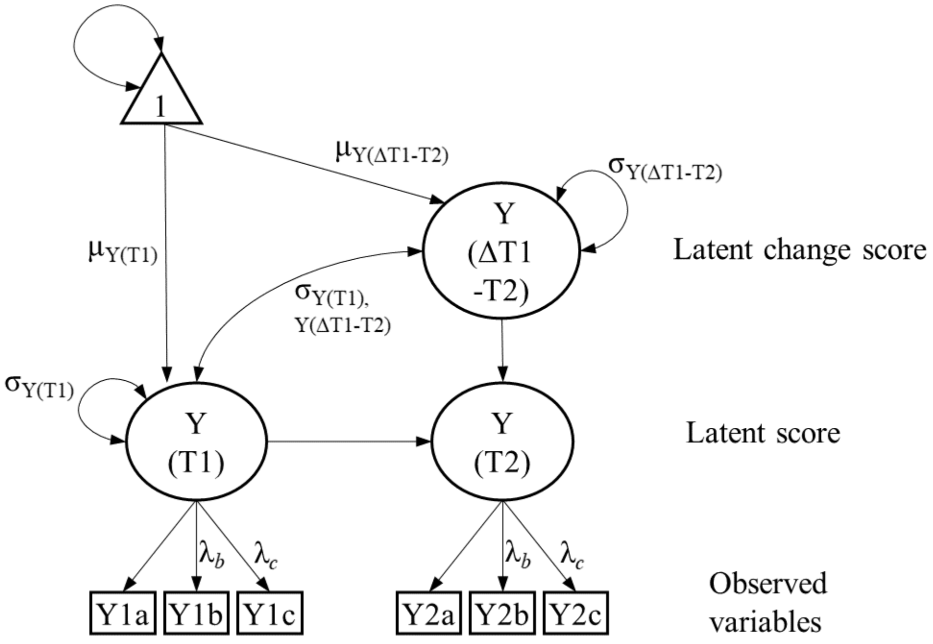

Figure S1. Description of the latent change score model across Time 1 (T1) and Time 2 (T2) with latent scores (i.e., factors) measured with three observed variables. Observed variables are represented by squares and latent constructs as circles. Unlabeled paths are fixed to 1.0. Labeled paths of λ_b and λ_c (i.e., factor loadings) are estimated but constrained equal (i.e., invariant) over time. While not included in the figure, residual variances and intercepts of observed variables are estimated and constrained equal over time and residual covariances among same observed variables across time are estimated. Mean of Y(T1) is set to zero for model identification.

Table S1. The estimates including the control variables for the models M2a-d, M3a-d, M5a-d, M6a-d, M8a-d, and M9a-d shown in Table 2. Standardized path estimates with 95% confidence intervals (CI) are shown. **Estimates with 95% CI excluding zero are bolded.** [T1=Time 1; T2= Time 2; T3=Time 3; T4=Time 4].

Model	Predictor variable	Standardized regression coefficient	95% CI	Standardized regression coefficient	95% CI	Standardized regression coefficient	95% CI	Standardized Regression coefficient	95% CI
		**Outcome variable: Burnout**							
		ΔT1-T2 (a)		ΔT2-T3 (b)		ΔT3-T4 (c)		ΔT1-T4 (d)	
M2a-d	Living alone	0.146	−0.064–0.356	0.088	−0.181–0.356	0.034	−0.215–0.284	0.195†	−0.033–0.423
	Age	−0.004	−0.013–0.005	**−0.018**	**−0.033–−0.003**	0.006	−0.004–0.015	−0.005	−0.016–0.006
	Gender	0.058	−0.141–0.257	0.199	−0.072–0.470	0.068	−0.146–0.282	0.173†	−0.030–0.375
	Education	−0.017	−0.144–0.109	−0.046	−0.220–0.127	−0.108	−0.248–0.033	−0.048	−0.187–0.091
	R^2	0.008	−0.007–0.023	**0.055**	**0.017–0.093**	0.014	−0.006–0.034	0.021†	−0.004–0.046
M3a-d	Telework	0.045	−0.218–0.308	−0.270†	−0.559–0.020	0.181	−0.090–0.451	−0.058	−0.303–0.187
	Age	−0.003	−0.014–0.008	**−0.023**	**−0.040–−0.007**	0.006	−0.005–0.017	−0.007	−0.019–0.006
	Gender	0.061	−0.167–0.290	0.041	−0.270–0.352	0.083	−0.157–0.324	0.090	−0.147–0.327
	Education	−0.109	−0.253–0.035	0.016	−0.185–0.218	−0.132	−0.312–0.049	−0.077	−0.231–0.078
	R^2	0.008	−0.009–0.025	**0.093**	**0.039–0.147**	0.016	−0.008–0.040	0.015	−0.009–0.039
		**Outcome variable: Job boredom**							
		ΔT1−T2 (a)		ΔT2−T3 (b)		ΔT3−T4 (c)		ΔT1−T4 (a)	
M5a-d	Living alone	**0.258**	**0.029–0.487**	0.118	−0.132–0.367	0.091	−0.165–0.346	**0.272**	**0.025–0.520**
	Age	0.005	−0.008–0.019	−0.008	−0.019–0.002	**−0.012**	**−0.024–−0.001**	−0.006	−0.017–0.005
	Gender	0.199	−0.043–0.441	0.035	−0.188–0.259	−0.116	−0.338–0.107	0.078	−0.137–0.294
	Education	0.079	−0.072–0.230	−0.079	−0.221–0.062	0.025	−0.121–0.172	0.063	−0.076–0.202
	R^2	**0.031**	**0.002–0.060**	0.016	−0.006–0.038	0.025†	−0.002–0.052	0.023†	−0.003–0.049
M6a-d	Telework	0.152	−0.150–0.454	0.116	−0.168–0.400	0.141	−0.143–0.425	0.192	−0.095–0.479
	Age	0.001	−0.016–0.019	−0.007	−0.019–0.005	**−0.015**	**−0.028–−0.002**	**−0.012**	**−0.023–−0.001**
	Gender	0.226	−0.083–0.536	−0.029	−0.278–0.221	−0.093	−0.351–0.166	0.073	−0.170–0.316
	Education	0.080	−0.114–0.274	−0.145	−0.321–0.031	−0.024	−0.208–0.160	0.045	−0.124–0.214
	R^2	0.026	−0.005–0.057	0.017	−0.008–0.042	0.034†	−0.001–0.069	0.031†	−0.003–0.063

		Outcome variable: Work engagement							
		ΔT1−T2 (a)		ΔT2−T3 (b)		ΔT3−T4 (c)		ΔT1−T4 (d)	
M8a-d	Living alone	−0.131	−0.327–0.064	−0.088	−0.319–0.143	0.139	−0.106–0.385	−0.031	−0.236–0.174
	Age	**0.010**	**0.001–0.019**	0.008	−0.002–0.017	0.002	−0.008–0.012	**0.010**	**0.001–0.018**
	Gender	0.075	−0.123–0.273	−0.079	−0.297–0.139	−0.084	−0.302–0.133	−0.162	−0.360–0.036
	Education	0.107†	−0.015–−0.015	−0.055	−0.192–0.082	0.085	−0.060–0.230	0.070	−0.060–0.201
	R^2	0.023†	−0.003–0.049	0.013	−0.006–0.032	0.010	−0.007–0.027	0.022†	−0.003–0.047
M9a-d	Telework	0.220†	−0.032–0.471	0.004	−0.270–0.278	−0.056	−0.303–0.190	0.197†	−0.013–0.407
	Age	0.008	−0.002–0.017	0.010†	−0.001–0.021	0.002	−0.009–0.012	0.008	−0.002–0.017
	Gender	0.073	−0.158–0.305	−0.231†	−0.467–0.005	−0.076	−0.303–0.151	**−0.289**	**−0.511–−0.067**
	Education	**0.151**	**0.002–0.301**	−0.091	−0.255–0.073	0.088	−0.077–0.252	0.056	−0.096–0.209
	R^2	**0.046**	**0.006–0.086**	0.034†	−0.001–0.069	0.005	−0.009–0.019	0.046	**0.006–0.086**

† Estimates with 90% confidence interval excluding zero are marked.

Δ Indicates within-person change across specific time span. Each predictor variable is estimated in separate models.
